# Improvement in multiple dimensions of fatigue in patients with fibromyalgia treated with duloxetine: secondary analysis of a randomized, placebo-controlled trial

**DOI:** 10.1186/ar3359

**Published:** 2011-06-13

**Authors:** Lesley M Arnold, Fujun Wang, Jonna Ahl, Paula J Gaynor, Madelaine M Wohlreich

**Affiliations:** 1 Women's Health Research Program, Department of Psychiatry and Behavioral Neuroscience, University of Cincinnati College of Medicine, 222 Piedmont Avenue, Suite 8200, Cincinnati, OH 45219, USA; 2 Statistics, MedImmune, LLC, 35 W. Watkins Mill Road, Gaithersburg, MD 20878, USA; 3 Scientific Communications, Lilly USA, LLC, Drop Code 4125, Indianapolis, IN 46285, USA; 4 Clinical Research, Lilly USA, LLC, Drop Code 4125, Indianapolis, IN 46285, USA

## Abstract

**Introduction:**

Fatigue is one of the most disabling symptoms associated with fibromyalgia that greatly impacts quality of life. Fatigue was assessed as a secondary objective in a 2-phase, 24-week study in outpatients with American College of Rheumatology-defined fibromyalgia.

**Methods:**

Patients were randomized to duloxetine 60-120 mg/d (*N *= 263) or placebo (*N *= 267) for the 12-week acute phase. At Week 12, all placebo-treated patients were switched to double-blind treatment with duloxetine for the extension phase. Fatigue was assessed at baseline and every 4 weeks with the Multidimensional Fatigue Inventory (MFI) scales: General Fatigue, Physical Fatigue, Mental Fatigue, Reduced Activity, and Reduced Motivation. Other assessments that may be associated with fatigue included Brief Pain Inventory (BPI) average pain, numerical scales to rate anxiety, depressed mood, bothered by sleep difficulties, and musculoskeletal stiffness. Treatment-emergent fatigue-related events were also assessed. Changes from baseline to Week 12, and from Week 12 to Week 24, were analyzed by mixed-effects model repeated measures analysis.

**Results:**

At Week 12, duloxetine versus placebo significantly (all p < .05) reduced ratings on each MFI scale, BPI pain, anxiety, depressed mood, and stiffness. Improvement in ratings of being bothered by sleep difficulties was significant only at Weeks 4 and 8. At Week 24, mean changes in all measures indicated improvement was maintained for patients who received duloxetine for all 24 weeks (*n *= 176). Placebo-treated patients switched to duloxetine (*n *= 187) had significant within-group improvement in Physical Fatigue (Weeks 16, 20, and 24); General Fatigue (Weeks 20 and 24); Mental Fatigue (Week 20); and Reduced Activity (Weeks 20 and 24). These patients also experienced significant within-group improvement in BPI pain, anxiety, depressed mood, bothered by sleep difficulties, and stiffness. Overall, the most common (> 5% incidence) fatigue-related treatment-emergent adverse events were fatigue, somnolence, and insomnia.

**Conclusions:**

Treatment with duloxetine significantly improved multiple dimensions of fatigue in patients with fibromyalgia, and improvement was maintained for up to 24 weeks.

**Trial registration:**

ClinicalTrials.gov registry NCT00673452.

## Introduction

Fibromyalgia is a chronic pain disorder that has been estimated to affect as many as 5 million individuals in the US, most of whom are women [1]. In addition to widespread pain, symptoms that may include sleep disturbances, fatigue, depression, anxiety, and problems with memory and concentration characterize fibromyalgia [2-4]. Among these, fatigue greatly impacts quality of life and has been identified as one of the most disabling symptoms associated with fibromyalgia [4]. Individuals with fibromyalgia report that their fatigue typically is not alleviated by sleep or rest [5] but is a physical tiredness, and these people have low energy and require increased effort to overcome inactivity and perform physical tasks [4,6]. Patients with fatigue report having decreased mental endurance and slowed thinking and feel overwhelmed [4]. Symptoms of fibromyalgia that may contribute to fatigue include pain [6-8], stiffness [8], sleep quality [6-9], and depression [6,7,10].

Medications currently approved for the management of fibromyalgia include duloxetine hydrochloride (hereafter referred to as duloxetine), pregabalin, and milnacipran. Duloxetine is a potent serotonin and norepinephrine reuptake inhibitor that has been approved by the US Food and Drug Administration for treatment of major depressive disorder (MDD) and generalized anxiety disorder (GAD) and for the management of pain associated with diabetic peripheral neuropathy, management of chronic musculoskeletal pain, and management of fibromyalgia. In past trials in fibromyalgia, the efficacy of duloxetine versus placebo on improvement in secondary measures of fatigue has not been consistent. Two of the fibromyalgia trials assessed fatigue as a secondary outcome by using the Multidimensional Fatigue Inventory (MFI) [11], which measures multiple domains of fatigue on five scales: General Fatigue, Mental Fatigue, Physical Fatigue, Reduced Activity, and Reduced Motivation. One of the studies reported significant between-treatment differences in only Mental Fatigue at the end of 6 months of treatment with duloxetine 60 to 120 mg given once daily (QD) [12]. The other study reported significant between-treatment differences in improvement with duloxetine 60 mg QD in Reduced Motivation at the 12-week endpoint and in Mental Fatigue at both the 12- and 24-week endpoints. In the same study, treatment with duloxetine 120 mg QD compared with placebo was associated with significant improvement in Reduced Motivation by 12 weeks and in Physical Fatigue, Mental Fatigue, Reduced Motivation, and Reduced Activity after 6 months of treatment [13].

More recently, treatment with duloxetine 60 to 120 mg QD for 12 weeks in comparison with placebo was found to significantly improve fatigue on each MFI domain [14]. In this secondary analysis, we report monthly changes in fatigue domains and in symptoms that may be related to or may contribute to fatigue, such as pain, depressed mood, anxiety, sleep, and stiffness across the entire study. In addition, changes in MFI scales were assessed in subgroups of patients who were pain responders, those who reported 'feeling much better', and those who required a dose escalation in the acute phase. The current analyses were performed to better characterize improvement in fatigue during the entire 24 weeks of the study.

## Materials and methods

Details of the 12-week acute phase of the study (F1J-US-HMGB; trial registration NCT00673452) have been published [14]. Briefly, this was a 24-week, multicenter, randomized, double-blind, placebo-controlled trial in outpatients who were at least 18 years old and who had fibromyalgia as defined by the American College of Rheumatology. The purpose of the trial was to confirm the efficacy of flexibly dosed duloxetine 60 to 120 mg QD on patient-rated improvement in fibromyalgia. Double-blind dose adjustments via an interactive voice response system were allowed for patients who were not responding. Response was defined as an at least 50% reduction in pain as assessed by the Brief Pain Inventory (BPI) [15] 24-hour average pain item (referred to hereafter as BPI average pain). At weeks 4 and 8, non-responding patients in the duloxetine group had their dose increased from 60 to 90 mg QD. At week 8, patients who were not responding to 90 mg had their dose increased to 120 mg QD. If the patient could not tolerate the dose increase, it was reduced to the pre-escalation dose. After week 12, all patients remained on their current dose of duloxetine for the remainder of the study. Patients in the placebo group were transitioned to double-blind active treatment with duloxetine 60 mg QD after week 12.

Efficacy measures in this analysis were assessed at each study visit, which occurred every 4 weeks, and included the MFI, BPI average pain, and numerical rating scales assessing anxiety, mood, 'bothered by sleep difficulties', and musculoskeletal stiffness and Patient Global Impression of Improvement (PGI-I) [16]. The MFI scales each rate symptoms from 4 (low) to 20 (high). The BPI average pain item assesses pain with ratings from 0 (no pain) to 10 (pain as severe as you can imagine). The PGI-I is a categorical scale that patients use to rate their overall impression of how they are feeling since treatment began; ratings on the scale are as follows: 1 = very much better, 2 = much better, 3 = a little better, 4 = no change, 5 = a little worse, 6 = much worse, and 7 = very much worse. The numerical rating scales assessed patient-perceived severity of anxiety, mood, 'bothered by sleep difficulties', and musculoskeletal stiffness. These scales ranged from 0 ('not present/bothered by') to 10 ('extremely').

Response to treatment in the acute phase was defined as an at least 50% reduction from baseline in BPI average pain severity. Treatment-emergent adverse events were assessed for the incidence of events that might be associated with fatigue.

Statistical analyses were done on an intent-to-treat basis. All randomly assigned patients with a baseline visit and at least one post-baseline visit were included in the efficacy analyses, and all randomly assigned patients were included in the safety analyses. For the acute phase, these analyses included baseline to week 12. The extension-phase analyses used week 12 as the baseline and week 24 as the endpoint. All tested hypotheses were considered statistically significant if the two-sided *P *value was not more than 0.05 (unless otherwise specified). *P *values are provided where valid statistical inferences can be made.

A restricted maximum likelihood-based MMRM (mixed-effects model repeated measures) analysis was used on longitudinal changes from baseline for continuous efficacy measures. The model included the fixed categorical effects of treatment, investigator, visit, and treatment-by-visit interaction as well as the continuous, fixed covariates of baseline score and baseline score-by-visit interaction. An unstructured covariance matrix was used to model the within-patient errors. Significance tests were based on least-squares means and type III sum of squares. Efficacy results presented here are from the MMRM analysis unless otherwise noted. Last observation carried forward (LOCF) changes from baseline to endpoint were analyzed by using an analysis of covariance (ANCOVA) model with the terms of treatment, investigator, and baseline scores. The term 'mean' refers to the least-squares mean, which is the estimated mean from a specific model (MMRM or LOCF ANCOVA).

Subgroup analyses were conducted on acute-phase mean changes in MFI scale ratings in pain responders and non-responders, patients with endpoint PGI-I of not greater than 2 or greater than 2, and before and after dose escalations in the acute phase. The models included baseline, treatment, investigator, subgroup, and treatment-by-subgroup interaction. Statistical analyses were performed with SAS software (SAS Institute Inc., Cary, NC, USA).

## Results

A description of the patient population and the acute-phase results have been reported previously [14] and will be briefly summarized here. Overall, most of the patients (93.2% of 530) were women who were middle-aged (50.2 ± 11.1 years old) Caucasians (77.4%) or Hispanics (15.7%). About 18% of the study population had a diagnosis of comorbid current MDD, and about 8% had a diagnosis of comorbid current GAD. At the acute-phase baseline, patients reported having moderate to severe fatigue symptoms, moderately severe pain, and musculoskeletal stiffness and being bothered by sleep difficulties. Severity of anxiety and depressed mood was mild to moderate.

**Figure 1:**
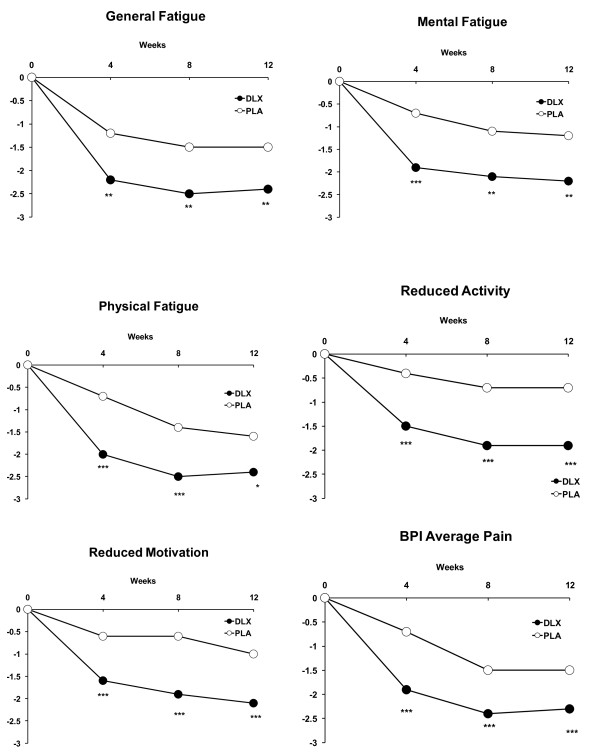
**Reduction (mean changes) from baseline in Multidimensional Fatigue Inventory domains and Brief Pain Inventory (BPI) average pain severity**. Comparisons versus placebo: *P ≤ 0.05, **P ≤ 0.01, ***P ≤ 0.001. DLX, duloxetine; PLA, placebo.

**Figure 2:**
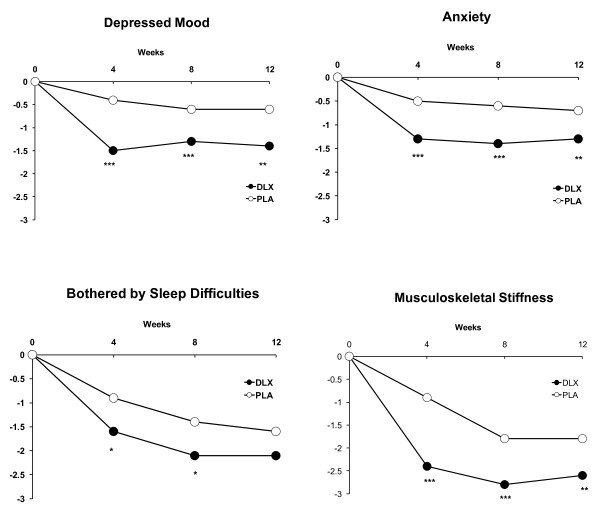
**Reduction (mean changes) from baseline in depressed mood, anxiety, 'bothered by sleep difficulties', and musculoskeletal stiffness**. Comparisons versus placebo: *P ≤ 0.05, **P ≤ 0.01, ***P ≤ 0.001. DLX, duloxetine; PLA, placebo.

In the acute phase, there was a statistically significant mean reduction (improvement) versus placebo on each MFI domain scale rating and BPI average pain measures at weeks 4, 8, and 12 (Figure 1). In addition, there was a statistically significant improvement versus placebo in patient ratings of anxiety, depressed mood, and musculoskeletal stiffness at weeks 4, 8, and 12 (Figure 2), but ratings of being bothered by sleep difficulties were significant at weeks 4 and 8 only. In both treatment groups, acute-phase mean reductions from baseline in MFI scale ratings in patients who were pain responders were 2 to 3 points lower on average (showing improvement in fatigue) compared with a less than 1 point decrease in patients who were non-responders (Table 1). Acute-phase mean changes from baseline in MFI scale ratings in patients with acute-phase endpoint PGI-I ratings of not greater than 2 were at least two to three times greater than the mean changes in patients with endpoint PGI-I ratings of greater than 2, regardless of treatment received (Table 2). Mean changes at endpoint in MFI scale ratings in patients who had a dose escalation are summarized in Table 3. A total of 122 patients not responding to duloxetine 60 mg were escalated to the 90 mg dose. Those patients who responded to the 90 mg dose (*n *= 59) experienced further reductions (improvement) across the MFI fatigue domains. Those patients who did not respond to the 90 mg dose and were escalated to the 120 mg dose (*n *= 63) experienced minimal improvement.

**Table 1:** Mean changes in Multidimensional Fatigue Inventory ratings in pain responders and non-responders

Multidimensional Fatigue Inventory	Responders		Non-responders	
	**Duloxetine** **(*n *= 140)**	**Placebo** **(*n *= 103)**	**Duloxetine** **(*n *= 105)**	**Placebo** **(*n *= 150)**
	**Mean change (SD)**	**Mean change (SD)**	**Mean change (SD)**	**Mean change (SD)**

General Fatigue	-3.4 (3.8)	-2.8 (3.4)	-0.7 (3.3)	-0.4 (2.8)
Mental Fatigue	-3.0 (4.4)	-1.8 (3.6)	-0.9 (3.6)	-0.5 (3.2)
Physical Fatigue	-3.1 (3.9)	-2.7 (3.2)	-0.6 (2.7)	-0.3 (2.9)
Reduced Activity	-2.6 (4.0)	-1.7 (3.6)	-0.2 (3.3)	0.2 (3.4)
Reduced Motivation	-2.7 (3.3)	-1.6 (3.3)	-0.6 (3.1)	-0.4 (3.2)

Pain response was defined as an at least 50% reduction from baseline in Brief Pain Inventory average pain severity. A last-observation-carried-forward analysis was conducted during the acute phase. SD, standard deviation.

**Table 2:** Mean changes from baseline in Multidimensional Fatigue Inventory ratings in patients with PGI-I of not more than 2 or greater than 2

Multidimensional Fatigue Inventory	PGI-I ≤ 2		PGI-I > 2	
	**Duloxetine** **(*n *= 114)**	**Placebo** **(*n *= 64)**	**Duloxetine** **(*n *= 132)**	**Placebo** **(*n *= 189)**
	**Mean (SE)**	**Mean (SE)**	**Mean (SE)**	**Mean (SE)**

General Fatigue	-3.8 (0.4)^a^	-4.2 (0.5)^a^	-0.9 (0.3)^a^	-0.4 (0.2)
Mental Fatigue	-3.1 (0.4)^a^	-3.1 (0.5)^a^	-1.1 (0.3)^a^	-0.5 (0.3)
Physical Fatigue	-3.6 (0.4)^a^	-3.7 (0.5)^a^	-0.8 (0.3)^a^	-0.7 (0.3)^a^
Reduced Activity	-3.2 (0.4)^a^	-3.0 (0.5)^a^	-0.2 (0.3)	0.2 (0.3)
Reduced Motivation	-2.9 (0.4)^a^	-2.9 (0.5)^a^	-0.7 (0.3)^a^	-0.1 (0.3)

^a^Statistically significant (*P *≤ 0.05) within-group change from baseline to endpoint at acute-phase endpoint (week 12). A last-observation-carried-forward analysis was conducted. PGI-I, Patient Global Impression of Improvement; SE, standard error.

**Table 3:** Multidimensional Fatigue Inventory ratings in patients who were escalated to a higher duloxetine dose

Multidimensional Fatigue Inventory	Duloxetine 90 mg (*n *= 59)^a^		Duloxetine 120 mg (*n *= 63)^b^	
	**Rating before escalation**	**Endpoint**	**Rating before escalation**	**Endpoint**
	**Mean (SD)**	**Mean change (SD)**	**Mean (SD)**	**Mean change (SD)**

General Fatigue	15.3 (3.4)	-0.7 (3.6)	16.0 (3.1)	-0.2 (3.0)
Mental Fatigue	12.1 (4.6)	-0.9 (3.0)	11.7 (4.0)	0.1 (3.3)
Physical Fatigue	14.6 (3.9)	-1.3 (3.7)	14.2 (3.6)	0.1 (2.6)
Reduced Activity	12.8 (4.3)	-1.0 (3.5)	12.8 (4.2)	0.2 (3.1)
Reduced Motivation	11.4 (3.9)	-1.1 (3.3)	11.5 (3.5)	-0.1 (3.1)

^a^Patients who responded to duloxetine 90 mg QD. ^b^Patients who did not respond to the 90 mg dose and were escalated to 120 mg. A last-observation-carried-forward analysis was conducted at acute-phase endpoint (week 12). SD, standard deviation.

At the end of the acute phase, 363 patients entered the 12-week extension phase, and all of them received double-blind treatment with duloxetine. Patients who received duloxetine in the acute phase continued on their stable dose of 60, 90, or 120 mg QD in the extension phase and were referred to as the duloxetine/duloxetine group (*n *= 176). Patients in the placebo group (*n *= 187) who continued in the extension phase received duloxetine 60 mg QD and were referred to as the placebo/duloxetine group. Extension-phase baseline (week 12) and mean changes at study endpoint (week 24) on each secondary measure are summarized in Table 4. For patients with 24 weeks of treatment with duloxetine, there were continued statistically significant within-group improvements in MFI General Fatigue and Reduced Motivation, BPI average pain, and patient ratings of anxiety, depressed mood, 'bothered by sleep difficulties', and musculoskeletal stiffness. Placebo patients who were transitioned to duloxetine also experienced improvement in each measure, and after 12 weeks of treatment there were statistically significant within-group improvements observed for all but MFI Mental Fatigue and Reduced Motivation.

**Table 4:** Extension-phase baseline (week 12) to week 24 changes in fatigue and related symptoms

Assessment	Duloxetine/Duloxetine (*n *= 176)		Placebo/Duloxetine (*n *= 187)	
	**Baseline mean (SD)**	**Mean change (SE)**	**Baseline mean (SD)**	**Mean change (SE)**

Multidimensional Fatigue Inventory				
General Fatigue	14.6 (3.8)	-0.61 (0.3)^a^	15.5 (3.7)	-1.02 (0.3)^a^
Mental Fatigue	13.1 (3.9)	-0.43 (0.3)	13.8 (4.2)	-0.32 (0.3)
Physical Fatigue	10.6 (4.1)	-0.41 (0.2)	11.7 (4.3)	-0.51 (0.2)^a^
Reduced Activity	11.9 (4.3)	-0.29 (0.3)	12.8 (4.2)	-0.64 (0.3)^a^
Reduced Motivation	10.2 (3.9)	-0.71 (0.3)^a^	11.2 (3.8)	-0.49 (0.3)
BPI average pain	4.1 (2.3)	-0.56 (0.2)^a^	4.9 (2.4)	-0.66 (0.2)^a^
Anxiety	2.2 (2.5)	-0.43 (0.2)^a^	3.1 (2.8)	-0.45 (0.2)^a^
Mood	2.2 (2.6)	-0.54 (0.2)^a^	3.1 (2.7)	-0.83 (0.2)^a^
Sleep difficulties	4.6 (3.1)	-0.81 (0.2)^a^	5.1 (2.9)	-1.12 (0.2)^a^
Stiffness	4.4 (2.7)	-0.67 (0.2)^a^	5.2 (2.7)	-0.82 (0.2)^a^

^a^Significant (*P *≤ 0.05) within-group improvement, mixed-effects model repeated measures analysis. BPI average pain, Brief Pain Inventory [15] 24-hour average pain item; SD, standard deviation; SE, standard error.

There were no significant between-treatment differences in the occurrence of fatigue-related treatment-emergent adverse events during the acute phase of the study, and these events became less frequent during the extension phase (Table 5). The most common events were fatigue, insomnia, and somnolence.

**Table 5:** Fatigue-related treatment-emergent adverse events that occurred during the acute and extension phases

Treatment-emergent adverse events	Acute phase		Extension phase	
	**Duloxetine** **(*n *= 263)**	**Placebo** **(*n *= 267)**	**Duloxetine/Duloxetine (*n *= 176)**	**Placebo/Duloxetine** **(*n *= 187)**

Chronic fatigue syndrome	0	0.4	0	0
Fatigue	9.5	7.1	3.4	4.8
Insomnia	9.1	7.1	3.4	3.7
Lethargy	1.9	0.7	0	0.5
Sedation	2.3	1.1	2.3	2.1
Sluggishness	0.4	0	0	0
Somnolence	5.7	3.4	1.1	3.7

Values are presented as percentages.

## Discussion

Treatment of fatigue has become an important component of the overall management of fibromyalgia because it has been identified by patients as being particularly bothersome and contributes to reduced quality of life [4,17,18]. Assessing fatigue is possible with a single question; however, the type of response a patient gives would depend on the nature of the question and what kind of fatigue is being experienced by the patient at that time. The MFI provides more in-depth information across five domains, each of which has been validated against a single global fatigue question, such as the 'Tiredness' question on the Fibromyalgia Impact Questionnaire [19]. Each domain was significantly associated with this global fatigue question, and this supports the notions that fatigue is multidimensional and that different aspects of fatigue should be measured separately [20]. Because patients with fibromyalgia often report fatigue symptoms that are physical as well as mental in nature, using the MFI allowed us to examine the effect of duloxetine across several dimensions of fatigue.

In this study, mean MFI scale ratings at baseline were nearly twice as severe as those reported for healthy individuals in a large US population, whose ratings were all less than 9 points [21]. The severity of fatigue in the patients with fibromyalgia in the present study was clinically significant because each MFI domain rating was more than 3 points higher than those of healthy individuals [21]. Furthermore, the MFI domain ratings in these patients were as severe as those reported by others for fibromyalgia [20] as well as patients with other chronic diseases like chronic fatigue syndrome [21], Sjögren syndrome [22], and chronic low back pain [23] and cancer patients receiving radiation therapy [24].

Treatment with duloxetine versus placebo significantly improved fatigue across all of the MFI domains within 4 weeks and continued to improve at each visit thereafter, and improvement was maintained for up to 24 weeks. The magnitude of improvement across the MFI domains was similar (reduction of about 2 points each), and this suggests that treatment with duloxetine improves not only global fatigue symptoms but also both physical and mental aspects of fatigue. Across acute-phase treatment groups, patient global impression of feeling at least 'much better' was associated with a 2- to 3-point decrease in fatigue severity across MFI domains, suggesting that improvement in fatigue may be as important as improvement in pain in this patient population. Previous studies have suggested that there is an association between pain and fatigue. For instance, changes in pain and fatigue in patients with fibromyalgia have been found to be moderately correlated with patient ratings of feeling 'better' [25]. In addition, a review of studies in patients with various chronic pain disease states reported that fatigue decreases when pain improves [26]. Also, pain was noted to be a predictor of fatigue in patients with rheumatoid arthritis, osteoarthritis, or fibromyalgia [27], and individuals with greater pain severity report greater fatigue [23]. In the present study, improvement in fatigue across MFI domains was two to three times greater in patients who were pain responders compared with non-responders. In addition, duloxetine 90 mg QD was associated with further reduction in pain [14] and improvement across fatigue domains for those patients who responded to this dose.

Treatment with duloxetine was associated not only with reduction in pain and fatigue in this study but also with reduction in severity of anxiety, depressed mood, 'bothered by sleep difficulties', and musculoskeletal stiffness. Changes in these symptoms may have contributed to the improvement observed in fatigue because, across all of these measures, with the exception of 'bothered by sleep difficulties' for which the mean change at week 12 did not separate from placebo (*P *= 0.06), significant improvement was noted at weeks 4, 8, and 12. However, when LOCF analysis was used, 'bothered by sleep difficulties' reported in the primary analysis of this study was significantly improved with duloxetine treatment as compared with placebo (*P *= 0.05) [14]. Overall, these findings are consistent with a study that found that a moderate (30% to 50%) to substantial (> 50%) reduction in pain was associated with significant reductions in fatigue, sleep disturbance, depression, and anxiety in patients with fibromyalgia [28].

Several limitations to the present study may impact the interpretation of the results. First, a specified level of fatigue severity was not required for patients to be included in this study. In addition, the results of this study may not be generalizable to patients with some psychiatric comorbid disorders or unstable medical or comorbid pain disorders or to patients who were treatment-refractory or disabled, because patients with these conditions were excluded from the study. Lastly, the results of this study do not definitively show the relationship between fatigue and any of the other symptoms of fibromyalgia; this relationship requires further research.

## Conclusions

Fatigue is a common and often disabling symptom associated with fibromyalgia. The MFI is a measure that captures the multidimensional nature of fatigue that is experienced by patients with fibromyalgia. This secondary analysis provides evidence for the efficacy of duloxetine in improvements in multidimensional fatigue domains across 24 weeks of treatment.

## Abbreviations

ANCOVA: analysis of covariance; BPI: Brief Pain Inventory; GAD: generalized anxiety disorder; LOCF: last observation carried forward; MDD: major depressive disorder; MFI: Multidimensional Fatigue Inventory; MMRM: mixed-effects model repeated measures; PGI-I: Patient Global Impression of Improvement; QD: *quaque die *(once daily).

## Competing interests

In the past 12 months, LMA has received grants/research support from Eli Lilly and Company (Indianapolis, IN, USA), Pfizer Inc (New York, NY, USA), Cypress Bioscience, Inc. (San Diego, CA, USA), Boehringer Ingelheim (Ingelheim, Germany), and Forest Laboratories, Inc. (New York, NY, USA) and honoraria as a consultant to Eli Lilly and Company, Pfizer Inc, Cypress Bioscience, Inc., Boehringer Ingelheim, Forest Laboratories, Inc., Allergan (Irvine, CA, USA), Takeda (Osaka, Japan), UCB (Brussels, Belgium), Theravance (South San Francisco, CA, USA), AstraZeneca (London, UK), sanofi-aventis (Paris, France), and Grünenthal (Aachen, Germany). FW is a former employee and JA, PJG, and MMW are current employees of and stockholders in Lilly USA, LLC (Indianapolis, IN, USA).

## Authors' contributions

LMA, FW, JA, PJG, and MMW made substantive contributions to the analysis and interpretation of data, were involved in drafting the manuscript or revising it critically for important intellectual content or both, and gave final approval of the version to be published. All authors read and approved the final manuscript.
